# Targeting individual pain catastrophizing to reduce migraine-related disability: a randomized controlled proof-of-concept trial using an online CBT intervention

**DOI:** 10.1038/s41598-026-65414-7

**Published:** 2026-08-28

**Authors:** Janosch Fox, Charly Gaul, Nicolina Laura Peperkorn, Julia Ohse, Joshua Krutzki, Jona Körber, Youssef Shiban

**Affiliations:** 1 Department of Psychology, PFH Göttingen, Weender Landstraße 3-7, 37073 Göttingen, Germany; 2 Department of Psychiatry and Psychotherapy, University Medical Center Göttingen, Von- Siebold-Straße 5, 37075 Göttingen, Germany; 3 Medical Faculty, University of Duisburg-Essen, Hufelandstraße 55, 45147 Essen, Germany; 4 Headache Center Frankfurt, Dalbergstraße 2a, 65929 Frankfurt, Germany

**Keywords:** Migraine, Disability, Pain catastrophizing, Psychological, CBT, Fear-avoidance model, Diseases, Medical research, Neurology, Neuroscience, Psychology, Psychology

## Abstract

This mechanistic randomized controlled proof-of-concept trial (RCT) investigated whether a brief online Cognitive Behavioral Therapy (CBT)-based avatar intervention can reduce individual pain catastrophizing in individuals with migraine and examined whether reductions in individual pain catastrophizing are associated with decreased migraine-related disability. Migraine is a highly disabling disorder, yet the mechanisms underlying migraine-related disability remain poorly understood. The Fear Avoidance Model (FAM) and recent research suggest that pain catastrophizing is a key mediator linking migraine experience with disability. A total of *N* = 55 individuals with migraine and ≥ 4 migraine days/month were randomized to either an intervention group (receiving cognitive restructuring targeting individual pain catastrophizing statements), or an active control group (contradicting false statements about migraine). Assessments at baseline, post-intervention, and six-week follow-up included individual Pain Catastrophizing (PCS-3), Headache Impact Test (HIT-6), as well as migraine-related and clinical characteristics. Intervention was evaluated using linear mixed-effects model. Hierarchical regression was used to determine the predictive effect of reductions in pain catastrophizing on reductions in disability. The intervention group demonstrated significantly greater reductions in pain catastrophizing at post-intervention (*p* = 0.009), while group differences diminished by follow-up. Decreases in individual pain catastrophizing were associated with reductions in migraine-related disability in both groups, explaining 24% of the variance (*R*^2^_*adj*_ = 0.24; *p* = 0.004). These findings suggest pain catastrophizing as a relevant factor in cognitive-affective pain processing contributing to migraine-related disability, as proposed by the FAM. It represents a potential therapeutic target that can be addressed through CBT-based interventions.

## Introduction

### Migraine

Migraine is a common and disabling primary headache disorder, affecting approximately 14% of the global population^1^. It has a profound impact on professional, academic, social, and family life and is the second leading cause of disability-adjusted life years worldwide^1^. Emerging evidence increasingly supports a multifactorial understanding of migraine, involving not only (neuro-)biological mechanisms, but also psychological and social factors, which may contribute to both disease progression and disability^2^.

### Migraine-related disability

While headache-related disability is recognized as an independent and clinically meaningful outcome^3–6^, the mechanisms underlying migraine-related disability remain underexplored. Empirical evidence suggests that attack frequency and other migraine characteristics contribute to disability; however they account for only a portion of the disability experienced by individuals^7–10^. Furthermore, disability may also change independently of attack frequency^11,12^, and greater disability is associated with increased direct and indirect disease-related costs, independent of headache frequency^13^. The International Classification of Functioning, Disability and Health (ICF) framework conceptualizes disability in general as the result of interactions between different health conditions and contextual factors^14^. Growing evidence highlights the significant role of psychological factors in exacerbating migraine-related disability, e.g., see^15–17^, supporting a multifactorial perspective that integrates both biological and psychological determinants. However, a comprehensive explanatory model detailing the mechanisms underlying migraine-related disability is still lacking.

### Fear avoidance model (FAM)

The FAM^18–20^ provides a biopsychosocial framework for understanding the development of pain-related disability. According to the model, dysfunctional pain processing can lead to fear of pain, pain hypervigilance, avoidance behaviors, and increased depressive symptoms, which collectively contribute to the maintenance and exacerbation of disability. Pain catastrophizing, a maladaptive cognitive-affective response to pain, is understood as a central factor within this process.

Although originally developed for musculoskeletal pain, the FAM’s assumptions show promise for transdiagnostic applicability^21^. However, its application to primary headache disorders remains limited and has so far been examined primarily in cross-sectional studies. In addition, in contrast to back pain, pain in migraine occurs episodically and in attacks. Migraine attacks are more difficult to predict, and avoiding triggers can be both helpful and disabling. Collectively, this may complicate cognitive-affective pain processing and foster distinct patterns of avoidance, given that a broader range of triggers is involved^22^. Therefore, the applicability of the FAM to migraine requires further empirical investigation.

### Pain catastrophizing as a maladaptive cognitive-affective process

Originally described by Ellis^23^ as an irrational and pessimistic anticipation of future negative events, catastrophizing has since evolved into a broader cognitive construct with relevance across various clinical conditions. For example, Gellatly and Beck^24^ conceptualize catastrophizing as a maladaptive, transdiagnostic process contributing to a variety of both physical and mental health conditions. While the specific content of catastrophic thoughts may vary by condition, the underlying cognitive mechanisms are thought to constitute a shared vulnerability factor^24^.

In the context of pain, catastrophizing has been defined as a maladaptive cognitive response, leading to an exaggerated threat appraisal of one’s symptoms^25^. This not only involves negative appraisal of the threat value of pain symptoms or pain-related stimuli, but also heightened attentional focus on pain and ruminative or repetitive negative thinking^26–29^.

### Modifying maladaptive cognitions using avatar-based interventions

A well-established therapeutic approach to changing maladaptive cognitions is Cognitive Behavioral Therapy (CBT), particularly through cognitive restructuring^30^. A novel and promising approach for the modification of maladaptive cognitions are avatar-based interventions, which facilitate targeted restructuring of these cognitions through interaction with a human-like virtual agent. Empirical studies have demonstrated that such interventions can reduce symptom severity, enhance quality of life, and improve well-being across diverse clinical populations (e.g., see^31,32^). Specifically, CBT-based avatar interventions, employing cognitive restructuring techniques targeting maladaptive cognitions, have been proven effective in reducing both dysfunctional beliefs and clinical symptoms^33–36^.

### Research gap

The mechanisms underlying migraine-related disability remain poorly understood, particularly from a biopsychosocial perspective. Recent evidence suggests that psychological factors, as conceptualized by the FAM, are associated with increased levels of disability in primary headache disorders^21,37,38^, with pain catastrophizing playing a pivotal mediating role^9^. However, there is a notable lack of longitudinal studies that specifically target pain catastrophizing as a modifiable mechanism to reduce disability, representing a critical gap with theoretical and clinical implications.

### Aim of the present study

This randomized controlled proof-of-concept trial investigates the mechanistic role of pain catastrophizing as a maladaptive cognitive mechanism underlying disability in migraine. We hypothesize that (1) individual pain catastrophizing cognitions can be modified using a brief online CBT-based intervention and (2) reductions in individual pain catastrophizing are associated with reduced disability.

## Methods

This study reports the primary analysis of longitudinal data from a randomized controlled trial (RCT) (German Register of Clinical Trials, ID: DRKS00033893). Preliminary baseline data have been reported previously^37^. Data collection occurred between April 2024 and February 2025 via an online survey using the LimeSurvey platform (LimeSurvey GmbH. LimeSurvey: An Open Source survey tool. http://www.limesurvey.org).

To reduce potential sources of bias, standardized recruitment and assessment protocols were implemented, as well as predefined eligibility criteria and the use of validated self-report instruments. Study procedures and reporting adhere to the recommendations outlined in the Consolidated Standard of Reporting Trials (CONSORT) Statement 2010^39^.

### Recruitment

Participants were recruited online through the official websites of the Migraine Patients Organization Germany (Migräne Liga e.V.), the German Migraine and Headache Society (DMKG e.V.), and the Headache Center Frankfurt. Additionally, printed notices in clinics and medical practices were used to raise awareness of the study. Recruitment was further supported by targeted outreach on the social media platforms Instagram and Facebook.

### Inclusion and exclusion criteria

Inclusion criteria for this study required (1) provision of a written informed consent, (2) a reported physician-confirmed migraine diagnosis, and (3) an average frequency of at least four migraine attacks per month. The migraine diagnosis was validated by assessing the ICHD-3 criteria^40^. To ensure adequate comprehension of the study material and to minimize potential confounders, exclusion criteria included current drug or alcohol misuse, uncorrected visual or hearing impairments, psychotic symptoms, or cognitive impairments that could negatively affect data acquisition.

### Ethics

All procedures involving human participants complied with the Declaration of Helsinki. Ethical approval was obtained from the Ethics Committee of the PFH Göttingen prior to data collection (December 21, 2023; reference number: 251981/7). Written informed consent was obtained from all participants. Prior to participation, participants received a written overview of the study’s objectives, the intervention, and the assessment procedures. They were further informed about data collection, storage, and protection, as well as their rights to access, delete, or restrict the processing of their personal data in accordance with GDPR regulations. No financial incentives were provided. This study was preregistered in the German Clinical Trials Register (DRKS) on March 15, 2024, under the DRKS-ID: DRKS00033893.

### Study design and procedure

**Fig. 1:**
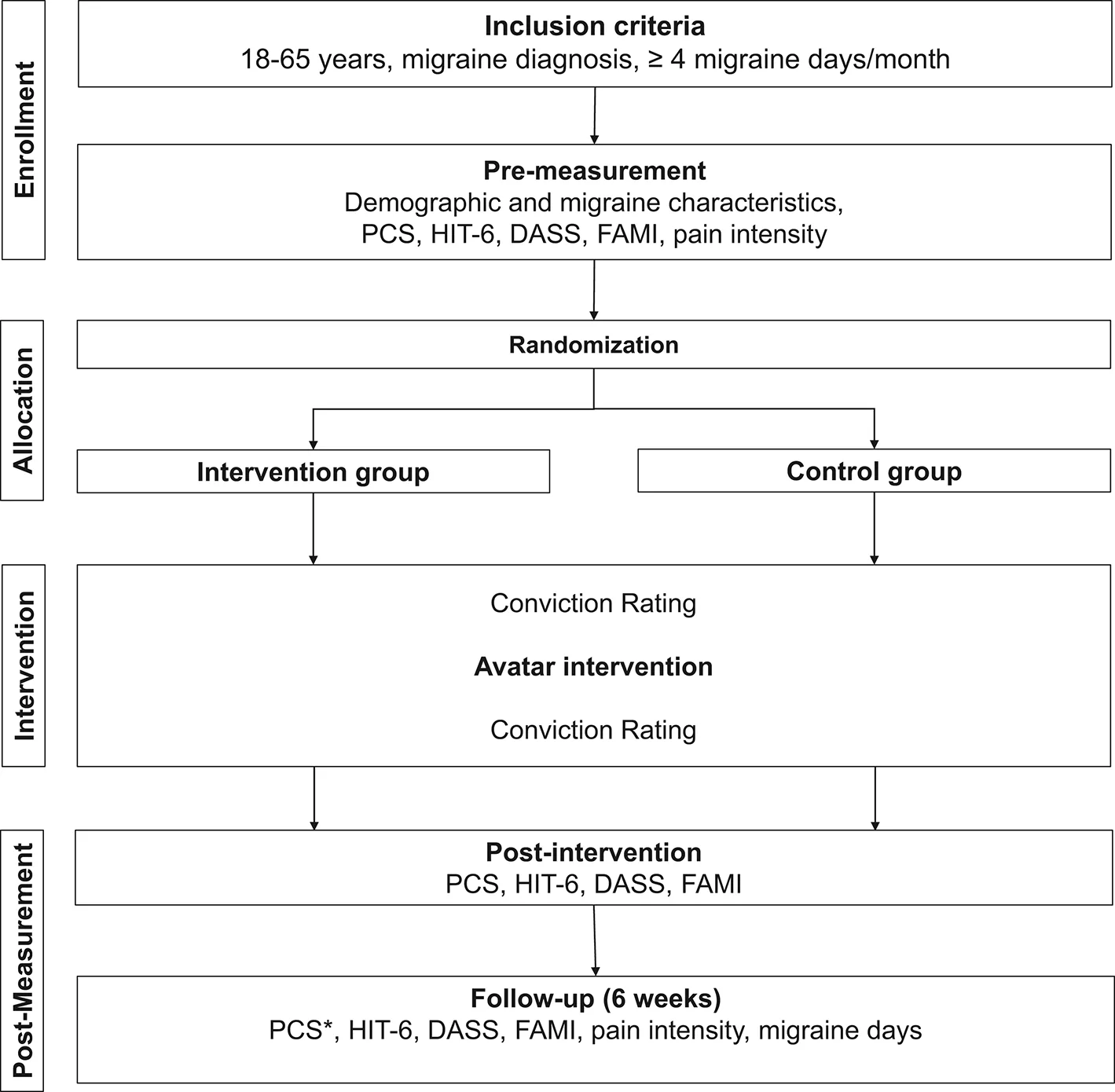
Study procedure. *Notes*: PCS, pain catastrophizing scale; HIT-6, headache impact test; DASS, depression anxiety and stress scales; FAMI, fear of attacks in migraine inventory.

This study was conceptualized as a parallel, randomized controlled trial (RCT) with assessments conducted at baseline, post-intervention, and follow-up, and was carried out online. The procedure is illustrated in Fig. 1. Participants were randomly assigned to either the intervention or control group. Randomization was automatically performed after completion of the baseline assessment using a simple random number generator implemented in LimeSurvey. Allocation was blinded, as participants were not informed of group allocation. The intervention comprised three sessions, followed by a post-intervention assessment and a follow-up assessment six weeks after the final session. Invitations to each session were sent via email. The intervention was fully automated, thereby preventing any potential influence from the study coordinators on the procedure or data collection.

### Intervention

**Fig. 2:**
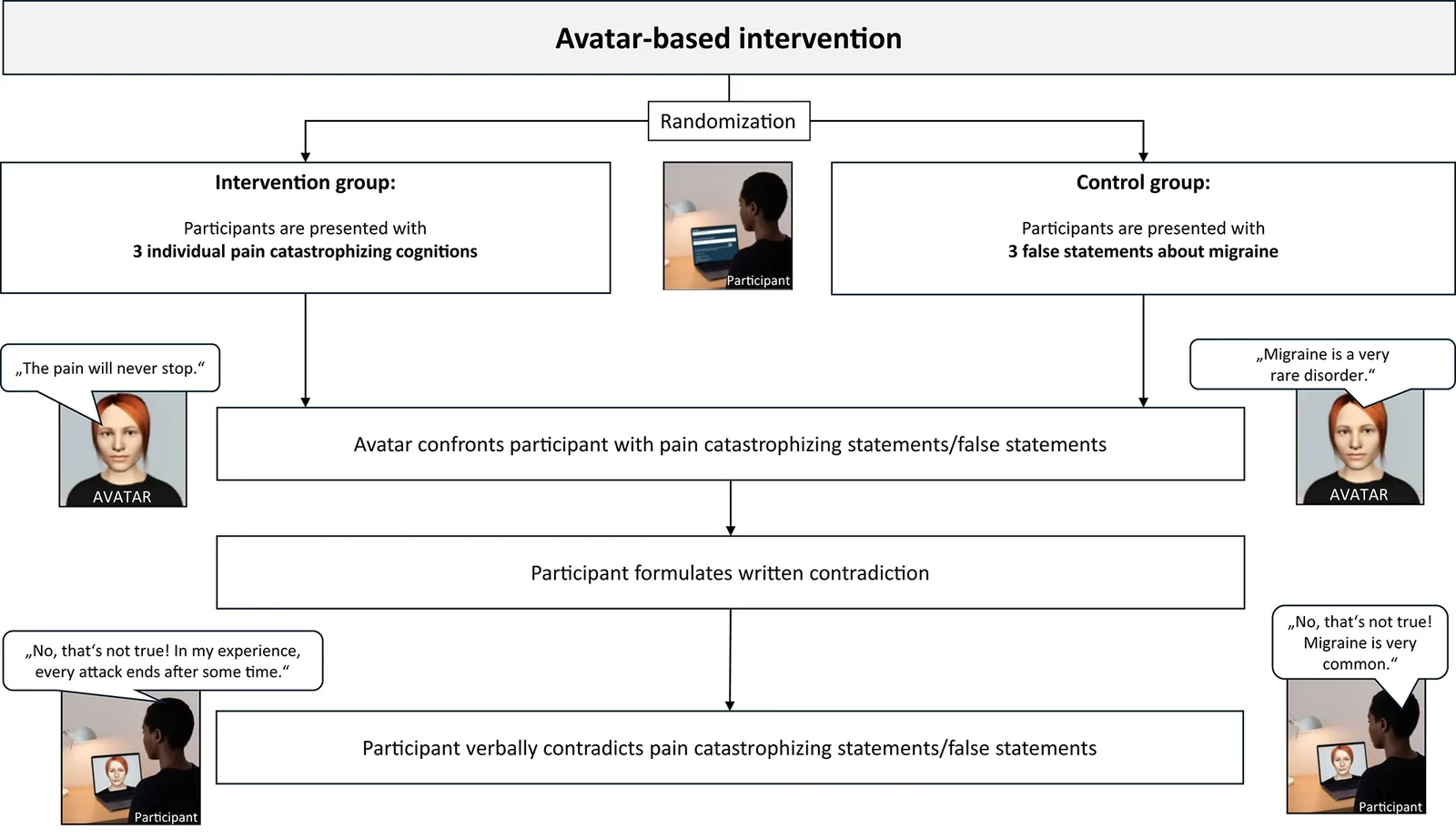
Interface and procedure of the intervention.

The intervention employed an idiographic approach and was based on cognitive-behavioral therapy. Cognitive restructuring was implemented, comprising the identification of dysfunctional cognitions, their confrontation, and the use of externalization as a therapeutic technique to facilitate their modification. Unlike traditional nomothetic approaches that target pain catastrophizing as a general construct, this intervention focused on the personalized identification and reduction of individual pain catastrophizing cognitions. The structure of the intervention was based on protocols previously established in the modification of maladaptive depressive, anxiety, and eating disorder cognitions^35,36,41,42^. Figure 2 illustrates the procedure.

The intervention was designed as a short-term program comprising three sessions. Participants in the intervention group were presented with individual pain catastrophizing statements. To generate these, each participant’s baseline responses on the Pain Catastrophizing Scale (PCS)^43^, an established instrument for assessing pain catastrophizing, were automatically analyzed in Lime Survey. The three highest-rated items per participant were identified (with random selection in case of ties) and were minimally reformulated in the second person (e.g., “The pain will never stop” became “Your pain will never stop”) to enhance personal relevance and engagement. The control group received an active control condition consisting of statements about migraine explicitly declared as false, designed to address and clarify common misconceptions (“Migraine is a very rare condition.“, “Migraine can manifest itself only through headaches and no other symptoms.“, “Migraine is a purely psychological disorder.“). Participants were instructed to contradict these statements by developing functional counterarguments and formulating a written objection. Subsequently, participants engaged in an interaction with the avatar, which vocalized the statements; participants were prompted to counter the avatar verbally, while their written contradictions were simultaneously displayed. Before and after each session, participants rated their level of conviction (0–100) regarding the statements presented by the avatar. In each session, participants received three sets, each containing three statements. The sessions lasted approximately 10 to 15 minutes, and participants were invited to attend sessions at intervals of three days.

The experiment was realized as a web-based application developed using the Unity3d engine and featured a virtual avatar created with Daz 3D’s Genesis 8 model. The avatar, a Caucasian-, female-looking figure, aged approximately 20–40 years, depicted from head to shoulders, was centrally positioned on the screen. Text-to-speech was enabled via Google Cloud Text-to-Speech API with talking animations realized using the SALSA LipSync v2 Suite. All sessions were fully automated and required a PC/laptop to conduct them.

### Measures

Sociodemographic and clinical data were collected. Sociodemographic variables included age and sex, with sex reported as male, female, or diverse. Migraine characteristics were assessed through self-report measures, including (a) the number of days with migraine attacks in the past four weeks and (b) the average number of migraine days per month over the past three months. Participants also reported the presence of accompanying symptoms such as aura, nausea, and photophobia/phonophobia. To further capture relevant clinical characteristics, a battery of standardized instruments was administered, assessing pain catastrophizing, psychological distress, pain intensity, fear of attacks, and migraine-related disability.

#### Individual pain catastrophizing (PCS-3)

To assess individual pain catastrophizing, we used the items of the Pain Catastrophizing Scale (PCS)^43^ as a basis to derive a reduced three-item index (PCS-3). The PCS-3 score was calculated by summing each participant’s three highest-rated PCS items, resulting in a range from 0 to 12. When multiple items were equally highly rated, three were selected at random. Higher scores indicate greater levels of pain catastrophizing.

#### Pain catastrophizing scale

The Pain Catastrophizing Scale (PCS)^43^ is a well-established instrument for measuring dysfunctional cognitive and affective responses to pain. It consists of 13 items, which are categorized into three subdomains: rumination, magnification, and helplessness. Participants rate each item on a 5-point Likert scale ranging from 0 (“not at all”) to 4 (“all the time”), resulting in a total score ranging from 0 to 52. Higher scores reflect greater levels of pain catastrophizing, with a total score of ≥ 30 being considered pathological, as this threshold corresponds to the 75th percentile among individuals with chronic pain^44^.

#### Conviction rating

To assess the level of conviction in pain catastrophizing statements or false statements about migraine, participants rated their conviction using a Visual Analog Scale (VAS) with a slider ranging from 0 (not at all convinced) to 100 (completely convinced).

#### Fear of attacks in migraine (FAMI)

The Fear of Attacks in Migraine Inventory (FAMI)^45^ is a recently developed self-report instrument designed to evaluate attack-related fear in individuals with migraine. It comprises 29 items, distributed across three subscales: fear of negative consequences, attention and anticipation, and fear-avoidance^45^. Each item is rated on a 5-point Likert scale, ranging from 1 (“strongly disagree”) to 5 (“strongly agree”). A cutoff score of ≥ 116 has been proposed by the developers^46^.

#### Depression, anxiety and stress scales (DASS)

The German version of the Depression, Anxiety and Stress Scales (DASS)^47,48^ is a self-report instrument designed to assess psychological distress across three dimensions: depression, anxiety, and stress. Each subscale consists of 7 items, rated on a 4-point Likert scale ranging from 0 (“does not apply to me at all”) to 3 (“applied to me very much). Scores for each subscale are summed. Higher scores indicate greater symptom severity, with suggested cut-off scores of 10 for depression, 6 for anxiety, and 10 for stress^47^. Notably, exclusion of somatic items makes the questionnaire suitable for evaluating psychological distress in patients with pain, as highlighted by its developers^48^.

#### HIT-6

The six-item Headache Impact Test (HIT-6) was developed as a screening tool for assessing the impact of headaches in clinical practice as well as in research^49^. It measures the impact of headaches on social functioning, role functioning, vitality, cognitive functioning, and psychological distress. For each of the six items, five answering options are provided (6 = “Never”; 8 = “Rarely”; 10 = “Sometimes”; 11 = “Very Often”; 13 = “Always”). The total score is calculated by summing the six item responses, resulting in a range of 36–78, with higher scores indicating a greater impact^50^. Scores ≤ 49 indicate “little or no impact”, scores between 50 and 55 represent “some impact”, scores from 56 to 59 reflect “substantial impact”, and scores of 60–78 denote “severe impact”^50^.

#### Pain intensity

Pain intensity was assessed by evaluating participants’ average and maximum pain intensity over the past four weeks. Due to the 4-week recall period of the measure, pain intensity was assessed only at baseline and at follow-up. For this, we used a VAS, as this is a widely used tool in migraine research for measuring participants’ pain^51^. Participants rated both their average and maximum pain intensity on a horizontal scale, ranging from 0 (“no pain”) to 10 (“unbearable pain”). Higher scores reflect greater perceived pain severity.

### Statistical analysis

All statistical analyses were conducted using SPSS (version 28; IBM Corp.) or R (version 4.4.1)^52^ with the packages dplyr^53^, tidyr^54^, haven^55^, gtsummary^56^, glue^57^, MuMIn^58^, lme4^59^, lmerTest^60^, and emmeans^61^. Descriptive statistics summarizing the study population and outcome variables included measures of central tendency, dispersion, and frequency distributions. Prior to analysis, data for demographic and clinical variables were inspected for outliers to minimize measurement error, with no implausible values detected.

Between-group differences were assessed using independent *t*-tests or Mann-Whitney *U* tests for continuous variables and Chi-square tests for categorical variables. Within-group changes were analyzed using repeated measures ANOVA, independent *t*-test or paired Wilcoxon signed-rank test. To examine potential systematic differences between completers and non-completers, a dropout analysis was performed, testing for differences in sociodemographic and clinical baseline variables.

To evaluate the intervention, a 2 × 3 repeated measures ANCOVA was employed with the PCS-3 score as the dependent variable, group as the between-subject factor, and age, sex, and baseline depressiveness as covariates. To account for the substantial dropout in our study, a linear mixed-effects model (LMM) was conducted as sensitivity analysis. The LMM included fixed effects for group, timepoint, and their interaction (group × timepoint). Participant age, sex, and depressiveness were included as covariates. Random intercepts were specified for participants to account for repeated measures and interindividual differences. Based on the method proposed by Nakagawa and Schielzeth^62^, the variance explained by the linear mixed-effects model was quantified using marginal and conditional *R*^2^ values. These metrics reflect the proportion of variance explained by fixed effects alone (*R*^2^ *marginal*) and by both fixed and random effects combined (*R*^2^ *conditional*). Following the recommendations by Rights and Sterba^63^, marginal *R*^2^ values in psychological research using multilevel models are typically small to moderate. Values of *R*^2^_*marginal*_ > 0.30 therefore indicate meaningful explanatory power of fixed effects. The conditional *R*^2^ reflects the proportion of variance explained by the full model, including both fixed and random effects. However, *R*^2^_*conditional*_ incorporates variance components that are not the primary focus of hypothesis testing and are sample-dependent. Therefore, the marginal *R*^2^ was considered the primary indicator of model fit for evaluating the explanatory power of the fixed effects.

To evaluate whether changes in pain catastrophizing predicted changes in migraine-related disability, a hierarchical multiple linear regression was conducted. In model 1, sex, age, change in attack frequency, and change in depressive symptoms were entered as control variables. In model 2, change in pain catastrophizing was added as the predictor of interest. Change scores were computed by subtracting baseline values from follow-up values, such that negative scores indicate a reduction over time. The change in explained variance (*ΔR*^2^) between the models was used to determine whether the additional predictors significantly improved the model’s explanatory power. Derived from Cohen’s guidelines for *f*^*2*^, the corresponding *R*^*2*^ values for effect sizes are considered as follows: an *R*^*2*^ of 0.02 is considered a small effect, 0.13 a medium effect, and 0.26 a large effect. A two-sided alpha level of 0.05 was considered statistically significant for all analyses.

### Power analysis

A priori power analysis was conducted using G*Power 3.1.9.7 to determine the required sample size for a 2 × 3 repeated measures ANCOVA with group allocation (IG vs. CG) as the between-subjects factor and time (pre vs. post vs. follow-up) as the within-subjects factor. Assuming a small-to-moderate effect size (Cohen’s *f* = 0.18) based on previous findings^64,65^, a two-sided α of 0.05, and a desired power of 0.80, the analysis indicated that a total sample size of *N* = 54 participants was required. For the multiple linear regression examining whether changes in pain catastrophizing predicted changes in migraine-related disability, a minimum of *N* = 49 participants was required to detect a medium-to-large effect size (*f*^2^ = 0.30) with α = 0.05 and 80% power, based on previous research^21,37^. Based on previous work^35,36,66^, we anticipated a dropout rate of approximately 55%, resulting in a target sample size of *N* = 120.

## Results

### Study population

**Fig. 3:**
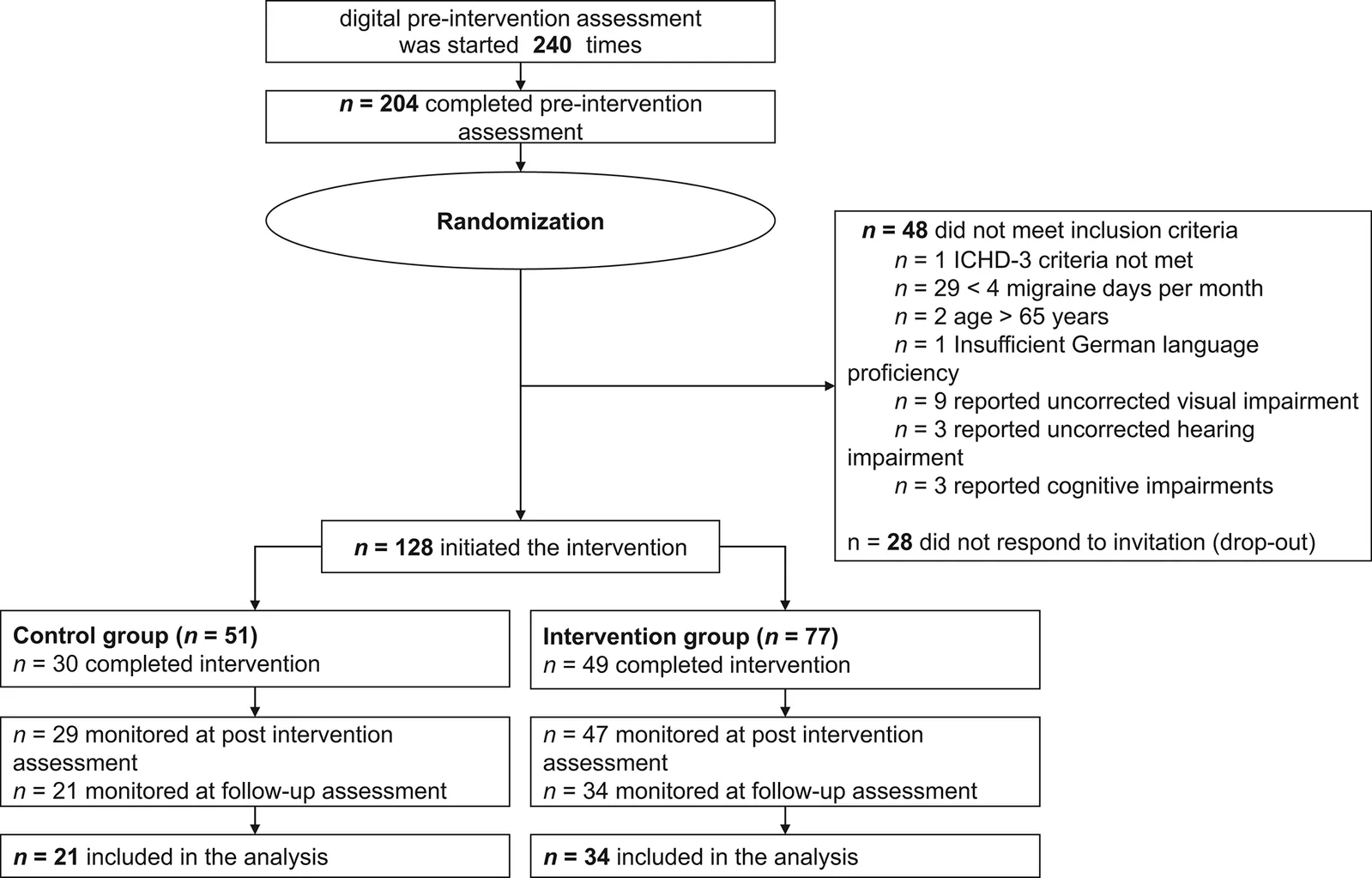
CONSORT participant flow diagram.

Of the 204 participants who completed the pre-intervention assessment, 76 had to be excluded due to not meeting inclusion criteria (mainly due to < 4 migraine days) or dropping out prior to the intervention. Of the remaining 128 participants who began the intervention, complete data was available for 66 participants post-intervention and for 55 participants at follow-up. Data inspection did not reveal implausible values. Figure 3 illustrates the study’s attrition process.

#### Drop-out analysis

A dropout analysis was conducted to examine potential systematic differences between participants who completed the full intervention (*n* = 55) and those who were randomized but did not complete it (*n* = 73). No significant differences (see Table 8 in the Appendix) were observed in terms of demographic, migraine-related or clinical characteristics (age (*p* = 0.853), sex (*p* = 0.706), pain catastrophizing (PCS-3: *p* = 0.652; PCS: *p* = 0.620), disability (*p* = 0.368), fear of attacks (*p* = 0.853), Headache Management Self-Efficacy (*p* = 0.579), depressive symptoms (*p* = 0.768), anxiety (*p* = 0.973), stress (*p* = 0.703), days with migraine attacks in the past 4 weeks (*p* = 0.753), or maximum and mean pain intensity (*p* = 0.414; *p* = 0.732).

#### Demographic and migraine-related characteristics of the sample at baseline

Table 1 presents the demographic and migraine characteristics of the study sample. The majority of participants indicated female sex (87.3%). The mean age of the sample was *M* = 36.1 years (*SD* = 11.4), ranging from 18 to 61 years. Eighteen participants (32.7%) reported a diagnosed psychiatric comorbidity, including *n* = 13 with depression and *n* = 5 with anxiety disorder. None of the participants indicated receiving psychotherapy at the time of intervention. No significant differences were observed between the groups in demographic or migraine-related baseline characteristics.

**Table 1:** Demographic and migraine characteristics of the sample at baseline, *N* = 55.

Characteristic	Total, *N* = 55	CG, *n* = 21	IG, *n* = 34	*p* between
Demographic characteristics				
Age, years); M (SD) [range]	36.1 (11.4) [18–61]	38.5 (10.8) [22–60]	34.6 (11.7) [18–61]	0.207
Sex; n (%)				
Female	48 (87.3)	19 (90.5)	29 (85.3)	0.575
Male	7 (12.7)	2 (9.5)	5 (14.7)	
Migraine-related characteristics				
Nausea; n (%)	36 (65.5)	14 (66.7)	22 (64.7)	0.882
Aura; n (%)	29 (52.7)	7 (33.3)	22 (64.7)	0.202
Photophobia/phonophobia; n (%)	49 (89.1)	19 (90.5)	30 (88.2)	0.796
Mean monthly migraine days in the past 3 months; Mdn (IQR)	6.00 (5.0–10.0)	7.0 (4.0-14.5)	5.50 (5.0–9.0)	0.510
Number of migraine days in the past 4 weeks; Mdn (IQR)	6.00 (5.0–9.0)	6.0 (3.0–9.0)	6.5 (5.0–9.0)	0.519
Number of migraine days in the past 4 weeks; n (%)				
4–7 days	34 (61.8)	11 (52.4)	23 (67.6)	0.427
8–14 days	12 (21.8)	5 (23.8)	7 (20.6)	
= 15 days	9 (16.4)	5 (23.8)	4 (11.8)	

CG, Control group. IG, intervention group.

#### Clinical characteristics

Between-group comparisons revealed no statistically significant differences at baseline across all clinical outcomes. Within-group analyses indicated significant improvements over time in both groups for pain catastrophizing, measured by PCS-3 and overall PCS, disability (HIT-6), and fear of attacks (FAMI). Furthermore, the intervention group exhibited significant increases in Headache Management Self-Efficacy (HMSE-G-SF) and significant reductions in depressive symptoms and stress (DASS), as well as maximum pain intensity and attack frequency, which were not observed in the control group. Table 2 provides a detailed overview of all the clinical characteristics of the sample.

Regarding pain catastrophizing, clinically relevant levels (PCS ≥ 30) were observed in 42.9% of participants in the control group and 32.4% in the intervention group at baseline. At post-intervention, these proportions decreased to 23.8% in the intervention group and 14.7% in the control group, with further reductions at follow-up to 14.3% and 11.8%, respectively.

**Table 2:** Clinical characteristics of the sample at baseline, post-intervention, and follow-up by group, *N* = 55.

Clinical characteristic	Group	Pre-intervention	Post-intervention	Follow-up	*p* within group	*p* between groups		
						Pre-intervention	Post-intervention	Follow-up
PCS-3, *M (SD)*	Control	9.1 (3.1)	7.5 (2.6)	6.0 (2.7)	**< 0.001**	0.561	0.052	0.838
	Intervention	9.6 (2.1)	6.1 (2.7)	5.8 (2.9)	**< 0.001**			
PCS, *M (SD)*	Control	25.0 (11.7)	22.0 (10.6)	18.9 (10.4)	**0.006**	0.726	0.502	0.998
	Intervention	26.1 (10.5)	20.0 (9.6)	18.9 (10.6)	**< 0.001**			
HIT-6, *M (SD)*	Control	67.5 (5.7)	65.5 (4.6)	62.8 (6.2)	**< 0.001**	0.215	**0.023**	0.340
	Intervention	65.7 (4.3)	62.5 (4.5)	61.1 (5.8)	**< 0.001**			
FAMI, *M (SD)*	Control	93.2 (23.5)	88.0 (23.5)	84.4 (24.7)	**0.026**	0.505	0.740	0.968
	Intervention	97.5 (22.7)	90.1 (21.4)	84.6 (22.3)	**< 0.001**			
DASS depression, *M (SD)*	Control	7.9 (5.0)	6.2 (4.2)	6.7 (3.7)	0.093	0.689	0.457	0.500
	Intervention	7.3 (5.8)	5.2 (5.5)	5.8 (5.6)	**0.016**			
DASS anxiety, *M (SD)*	Control	3.5 (2.5)	3.2 (3.4)	2.8 (3.6)	0.724	0.284	0.550	0.348
	Intervention	4.5 (4.3)	3.9 (5.1)	3.8 (4.6)	0.506			
DASS stress, *M (SD)*	Control	8.6 (4.1)	6.7 (3.8)	6.9 (3.5)	0.053	0.971	0.889	0.695
	Intervention	8.5 (4.2)	6.8 (4.4)	7.4 (4.9)	**0.023**			
Maximum pain intensity, *M (SD)*	Control	7.4 (1.8)	–	6.6 (2.7)	0.115	0.581	–	0.878
	Intervention	7.6 (1.6)	–	6.7 (1.9)	**0.002**			
Mean pain intensity, *M (SD)*	Control	5.3 (2.1)	–	4.5 (2.2)	0.053	0.438	–	0.138
	Intervention	5.7 (1.7)	–	5.4 (1.7)	0.280			
HMSE-G-SF, *M (SD)*	Control	24.0 (7.5)	26.6 (6.5)	24.2 (10.6)	0.133	0.402	0.093	0.096
	Intervention	25.7 (7.7)	29.6 (6.4)	28.8 (7.8)	**< 0.001**			

PCS-3, derived subscale of the PCS, calculated by summing each participant’s three highest-rated items: VAS, Visual Analog Scale; HIT-6, Headache Impact Test; DASS, Depression Anxiety and Stress Scales; PCS, Pain Catastrophizing Scale; FAMI, Fear of Attacks in Migraine Inventory; HMSE-G-SF, Headache Management Self-Efficacy Questionnaire (German Short Form); *SD*, standard deviation; *IQR*, interquartile range. Significant results in bold.

### Evaluation of the intervention

A 2 × 3 repeated measures ANCOVA was conducted to examine the effects of the intervention on pain catastrophizing (PCS-3) over time, controlling for sex, age, and depressiveness. Results of the 2 × 3 Repeated Measures ANCOVA are presented in Table 3. Between-subjects analyses revealed significant effects of sex (*p* = 0.017) and depressiveness (*p* < 0.001), whereas age (*p* = 0.960) and group (*p* = 0.745) did not emerge as significant predictors. Within-subjects effects showed a significant linear effect of time (*p* = 0.009) and a significant quadratic time × group interaction (*p* = 0.003), indicating that the intervention group exhibited a stronger reduction in PCS-3 scores from baseline to post, but not to follow-up. Estimated marginal means for PCS-3 at each time point are presented in Table 4.

**Table 3:** Results of the 2 × 3 repeated measures ANCOVA on PCS-3 scores.

Effects		*F*	*df*	*p*	$\eta_{p}^{2}$
Between-subjects effects					
Sex		6.045	1	**0.017**	0.108
Age		0.003	1	0.960	0.000
Group		0.107	1	0.745	0.002
Depressiveness		13.764	1	**< 0.001**	0.216
Within-subjects effects					
Time (linear)	Linear	7.475	1	**0.009**	0.130
Time (quadratic)	Quadratic	0.001	1	0.977	0.000
Time × group (linear)	Linear	0.436	1	0.512	0.009
Time × group (quadratic)	Quadratic	9.519	1	**0.003**	0.160

Significant results in bold.

**Table 4:** Estimated marginal means of PCS-3 across time and group in a 2 × 3 repeated measures ANCOVA.

Time	Group	PCS-3 Score, EMM [95%-CI]
Pre-intervention	Control	9.03 [8.05; 10.00]
	Intervention	9.66 [8.90; 10.42]
Post-intervention	Control	7.44 [6.37; 8.52]
	Intervention	6.11 [5.27; 6.95]
Follow-up	Control	5.76 [4.58; 6.94]
	Intervention	5.91 [4.99; 6.83]

EMM, Estimated marginal means.

#### Sensitivity analysis (linear mixed-effects model)

**Fig. 4:**
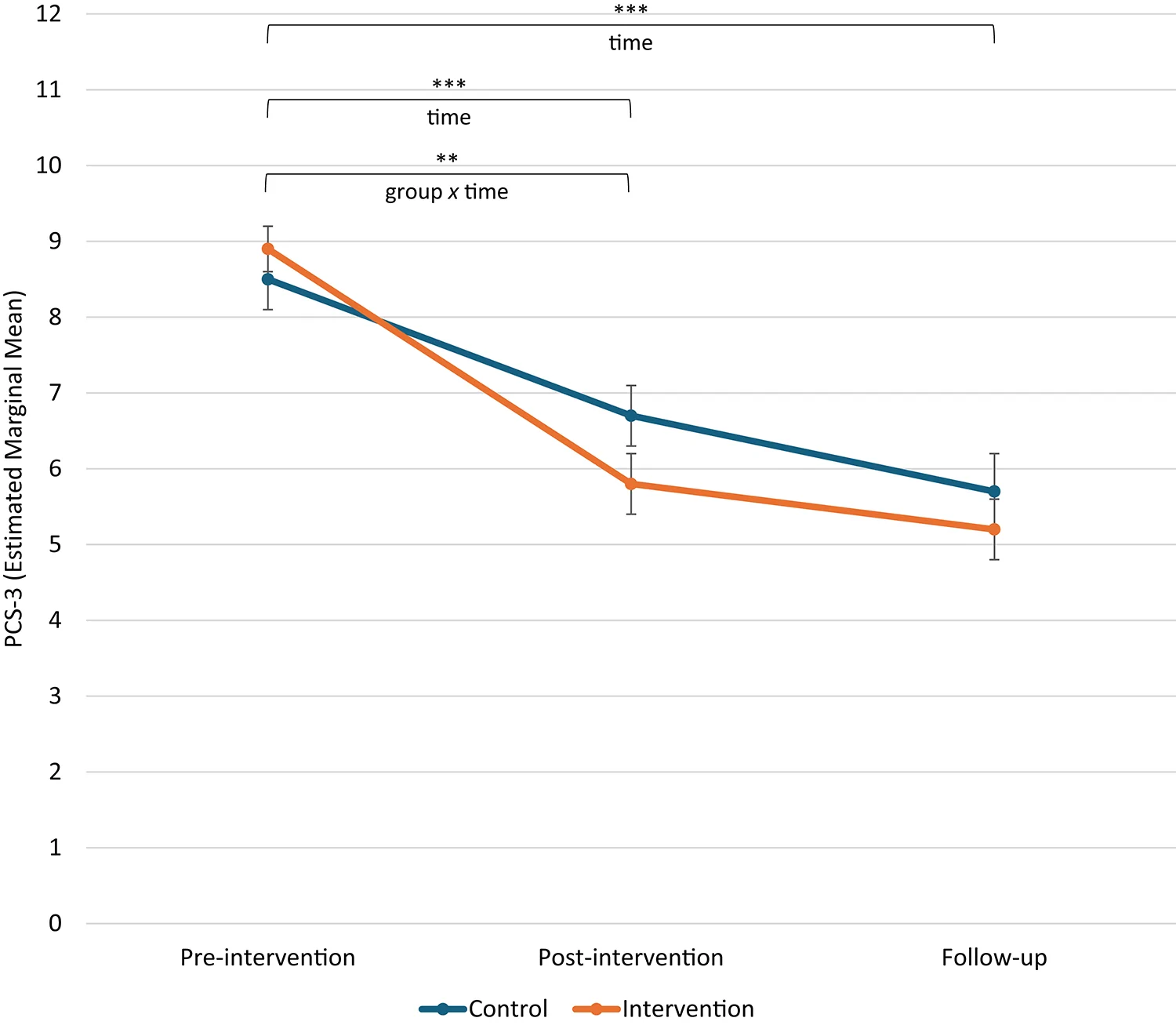
Trajectory of the model-based PCS-3-scores (LMM) of both groups throughout the intervention. *Notes*: Error bars represent ± 1 standard error.

As a sensitivity analysis, a linear mixed-effects model controlling for age, sex, and depressiveness confirmed a significant main effect of time, indicating a reduction in PCS-3 scores from baseline to post-intervention (*b* = − 1.76, *p* < 0.001) and from baseline to follow-up (*b* = − 2.78, *p* < 0.001). The group × time interaction was significant for the post-intervention comparison (*b* = − 1.37, *p* = 0.009), suggesting a significantly greater decrease in PCS-3 scores in the intervention group, but did not remain significant at follow-up (*p* = 0.101). Higher depressive symptoms were positively associated with PCS-3 scores (*p* < 0.001). The overall trajectory of PCS-3 scores is illustrated in Fig. 4.

Estimated marginal means (see Table 5) based on the LMM supported the trajectory of PCS-3 reduction over time in both groups, with no significant change in PCS-3 scores between the post- and follow-up timepoints (CG: *b* = 0.97, *p* = 0.084; IG: *b* = 0.44, *p* = 0.475).

**Table 5:** Estimated marginal means of PCS-3 scores by group and timepoint, based on the linear mixed model.

Timepoint	Group	M (SD)	SE	95%-CI
Pre-intervention (*n* = 91)	Control (*n* = 39)	8.45 (2.84)	0.47	[7.52, 9.38]
	Intervention (*n* = 52)	8.90 (2.37)	0.40	[8.10, 9.69]
Post-intervention (*n* = 87)	Control (*n* = 37)	6.70 (2.72)	0.47	[5.76, 7.63]
	Intervention (*n* = 50)	5.78 (2.65)	0.41	[4.97, 6.58]
Follow-up (*n* = 64)	Control (*n* = 26)	5.67 (2.78)	0.52	[4.64, 6.70]
	Intervention (*n* = 38)	5.16 (3.12)	0.44	[4.30, 6.02]

Significant results in bold.

The linear mixed-effects model explained 35.2% of the variance in PCS-3 scores by fixed effects alone (*R*^2^_*marginal*_ = 0.35) and 70.2% when including random effects (*R*^2^_*conditional*_ = 0.70).

#### Conviction ratings (manipulation check)

**Fig. 5:**
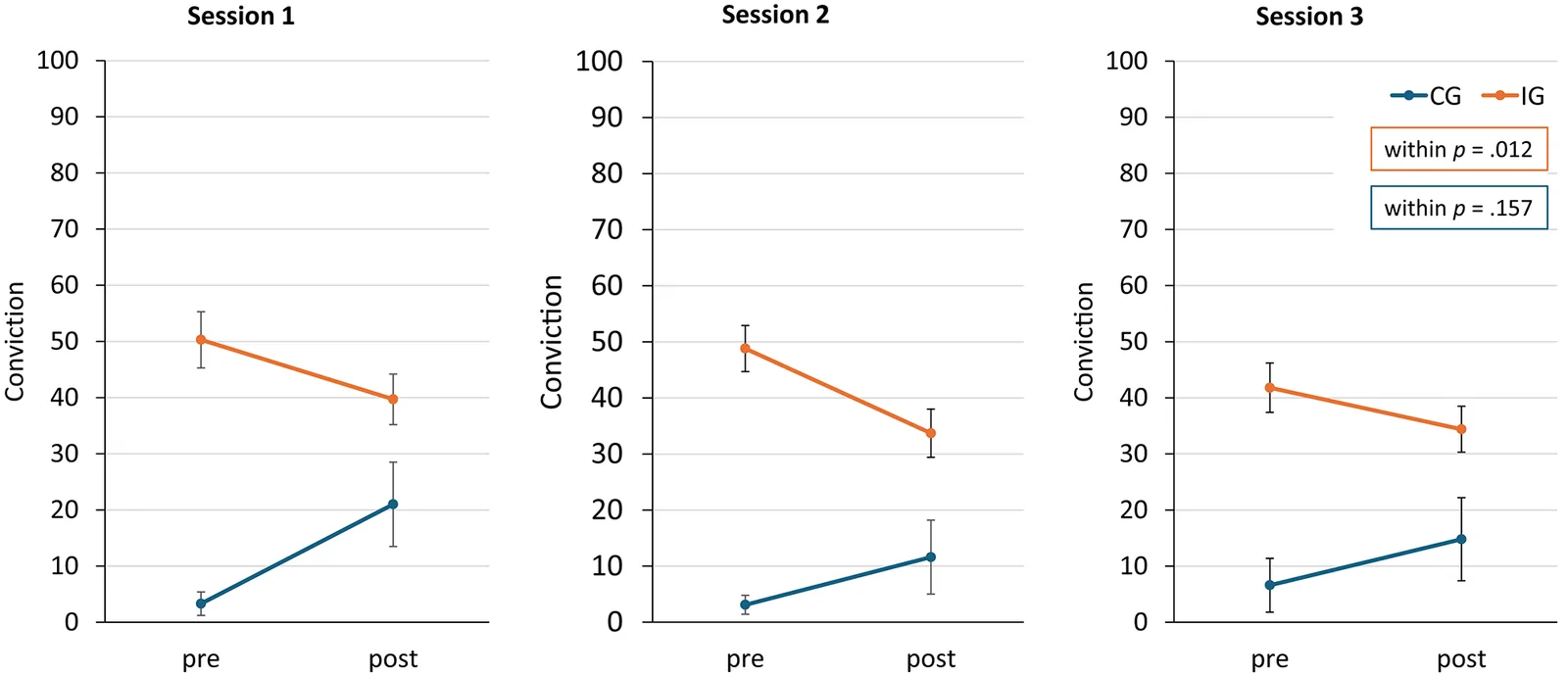
Session-wise mean conviction ratings of both groups across the three intervention sessions. *Notes*: Error bars represent ± 1 standard error (SE). CG, control group. IG, intervention group.

Conviction ratings of individualized pain catastrophizing statements (intervention group) or false facts about migraine (control group) were assessed before and after each of the three intervention sessions. In the intervention group, conviction ratings decreased significantly from pre- to post-assessment in all three sessions. In the control group, a significant increase was observed in Session 1, while changes in Session 2 and Session 3 were not statistically significant (see Table 6). Considering the overall intervention period, conviction ratings in the intervention group decreased significantly (*M* = − 15.83, 95% CI [− 28.02, − 3.65], *p* = 0.012), while no significant overall change was found in the control group (*M* = − 11.49, 95% CI [− 27.80, 4.82], *p* = 0.157). Figure 5 illustrates mean conviction ratings across the three sessions for both groups.

**Table 6:** Conviction ratings of individualized pain catastrophizing statements (intervention group) and false migraine-related statements (control group) assessed before and after each session.

Session	Group	Pre session, M (SD)	Post session, M (SD)	Δ pre-post (within)	*t* (*df*)	*p*
Session 1	Control	3.30 (9.57)	21.02 (34.25)	+ 17.72	− 2.37 (20)	**0.028**
	Intervention	50.27 (28.99)	39.71 (26.19)	− 10.56	2.22 (33)	**0.033**
Session 2	Control	3.14 (7.64)	11.60 (30.28)	+ 8.46	− 1.23 (20)	0.235
	Intervention	48.75 (24.10)	33.69 (24.79)	− 15.06	4.55 (33)	**< 0.001**
Session 3	Control	6.56 (22.13)	14.79 (34.13)	+ 8.23	− 0.89 (20)	0.386
	Intervention	41.83 (25.98)	34.44 (24.08)	− 7.39	3.41 (33)	**0.002**

Significant results in bold.

### Prediction of changes in migraine-related disability by changes in pain catastrophizing (PCS-3)

A hierarchical multiple linear regression model was conducted to examine whether changes PCS-3 predict changes in disability at follow-up, controlling demographics, depressiveness, and attack frequency. Results indicated that only change in PCS-3 was a significant predictor (see Table 7). The model explained 24% of the variance in disability (*R*^2^_*adj*_ = 0.24, *p* = 0.004), corresponding to a medium-to-large effect size.

**Table 7:** Hierarchical regression results: Change in pain catastrophizing (PCS-3) predicting change in migraine-related disability.

Model	*R*	*R* ^2^	*R* ^2^_adj_	*p*	Change in *R*^2^		ß	B	SE_B_	95%-CI	
					ΔR^2^_adj_	*p*				Lower bounds	Upper bounds
Model 1	0.39	0.15	0.07	0.141							
Intercept						**0.006**		10.656	3.690	3.147	17.984
Sex						0.295	− 0.144	− 2.346	2.215	− 6.800	2.109
Age						0.130	− 0.213	− 0.103	0.067	− 0.237	0.031
Δ Attack Frequency^a^						0.744	− 0.064	− 0.726	1.614	− 0.684	0.492
Δ Depressiveness^a^						0.068	0.256	0.321	0.172	− 0.024	0.666
Model 2	0.57	0.33	0.24	**0.004**	0.17	**0.001**					
Intercept						0.090		6.160	3.557	− 0.996	13.316
Sex						0.439	− 0.096	− 1.568	2.009	− 5.609	2.472
Age						0.188	− 0.168	− 0.081	0.060	− 0.202	0.041
Δ Attack frequency^a^						0.862	− 0.022	− 0.046	0.264	− 0.577	0.485
Δ Depressiveness^a^						0.180	0.171	0.215	0.158	− 0.103	0.532
Δ PCS-3^a^						**0.001**	0.436	0.928	0.267	0.392	1.465

^a^Change score pre-intervention to follow-up. Significant results in bold.

## Discussion

This RCT is one of the first theory-driven longitudinal studies examining pain catastrophizing as a modifiable cognitive pain processing mechanism underlying disability in migraine. This study investigated whether (1) pain catastrophizing can be reduced by a brief, online CBT-based intervention, and (2) whether a reduction in individual pain catastrophizing is associated with a reduction in migraine disability.

### Evaluation of intervention

The intervention was associated with a greater short-term reduction in pain catastrophizing compared to the active control group from baseline to post-intervention. This suggests a short-term effect rather than established effectiveness. Previous RCTs using similar methodological approaches have demonstrated that brief CBT-based online interventions can modify maladaptive cognitions in depressiveness^35^, eating disorders^33^, and social anxiety^67^. To our knowledge, only one longitudinal study^65^ has targeted pain catastrophizing in migraine using a CBT-based intervention delivered face-to-face. Similar to the present RCT, Thorn et al.^65^ demonstrated a reduction in pain catastrophizing, which was associated with improvements in clinical outcomes, including self-efficacy, a strong predictor of reduced disability^68,69^.

To evaluate the durability of intervention effects, outcomes were measured both immediately after the intervention and at a six-week follow-up, with no significant differences between groups observed at the six-week mark. Several explanations may account for this finding. First, the intensity of the intervention may have been insufficient. As found by Thorn et al.^65^, treatment duration appears to be a critical factor in the effectiveness of CBT-based interventions for modifying pain catastrophizing. Second, as the three highest-rated items at baseline were used to construct the primary outcome, regression to the mean represents a plausible explanation for the reductions observed in both groups. Third, the observed reduction in both groups at follow-up may reflect unintended therapeutic effects in the active control condition. Participants were instructed to counteract false information about migraine. Asking participants to refute false statements about migraine can itself induce cognitive restructuring, normalization, and stigma reduction. In addition, the educational component may have influenced cognitive beliefs by providing accurate information and reducing associated stigma. According to the FAM, threatening illness information is postulated to contribute to pain catastrophizing. Previous studies suggest that education-based interventions can improve primary outcomes in migraine^70^. Future research should consider increasing intervention dosage and investigate the (potentially positive) effects of education and de-stigmatization on both pain catastrophizing and disability.

### Findings in context of the FAM

Reductions in pain catastrophizing were associated with reductions in migraine-related disability, with moderate to large effect sizes that remained after adjusting for confounders such as attack frequency, depressive symptoms, and demographics. These findings are consistent with our hypothesis and the assumptions of the FAM. The FAM was originally developed in the context of back pain. The present results suggest that its relevance may extend to primary headache disorders characterized by episodic, attack-based pain. Specifically in migraine, emerging evidence indicate that pain catastrophizing appears to be a critical factor, functioning as a mediator between pain experience and disability^9,30^. While most previous studies have used cross-sectional designs, the present study provides longitudinal data on the association between changes in pain catastrophizing and changes in disability.

### Clinical implications

Pain catastrophizing emerged as an important and commonly reported factor in migraine. In this study, over one third of the population indicated pathological pain catastrophizing. As summarized by Amatrudo et al.^71^, cognitive restructuring techniques such as the use of thought records are effective tools for modifying maladaptive pain-related cognitions. By systematically identifying and reframing negative pain-related thoughts into more flexible and compassionate alternatives, such approaches can foster improved emotional regulation. The association between reductions in pain catastrophizing and reductions in migraine-related disability observed in our study points to pain catastrophizing as a potential diagnostic and therapeutic target.

### Limitations

This study has several strengths. It applied a rigorous, theory-driven longitudinal RCT design and implemented a standardized digital intervention to minimize confounding influences and potential biases. The intervention’s internal validity was further supported by manipulation checks, which showed significant reductions in conviction ratings of pain-catastrophizing statements and a lower proportion of participants exceeding pathological thresholds. Nevertheless, several limitations must be acknowledged. A key limitation of this study, as expected, is the high dropout rate. High dropout rates are common in online interventions without compensation^66,72^, and similar studies have reported comparable attrition^33,35^. To address this, we conducted linear mixed-effects modeling and dropout analyses, which revealed no evidence of systematic dropout. Future studies should include systematic assessments of intervention experience, including perceived acceptability, emotional responses, engagement, and potential sources of distress or disengagement during intervention sessions, to better understand reasons for attrition. Given the importance of therapeutic alliance in traditional CBT-based interventions, future avatar-based approaches may also benefit from considering alliance-related factors to enhance engagement, thereby improving adherence and sustaining effectiveness. Furthermore, the intervention in this study followed an idiographic approach to address individual pain catastrophizing. However, the use of a non-validated score limits the construct validity and generalizability to the broader construct of pain catastrophizing. Convergent validity analyses showed strong correlations between PCS-3 and total PCS scores at baseline (*r* = 0.87, *p* < 0.001), post-intervention (*r* = 0.80, *p* < 0.001), and follow-up (*r* = 0.80, *p* < 0.001). These findings suggest that the individualized three-item composite may approximate the broader catastrophizing construct across assessment points; however, this interpretation should be made with caution given the derived nature of the PCS-3 from the original PCS. Moreover, due to the automated online study workflow, eligibility verification and participant non-attendance occurred after randomization, resulting in unequal final group sizes. A further point is, that migraine attack frequency and disability were assessed via retrospective self-report questionnaires, making them potentially susceptibility to recall bias^73^. The debate about the validity of questionnaire vs. diary-based assessment is still ongoing^73–75^. However, this issue seems to be particularly important in pharmacological trials, where even small discrepancies in migraine days can affect study outcomes. In contrast, the present study focuses on the subjective disease burden. In this context, self-report measures are considered both appropriate and meaningful, as they capture the patient’s own experience of illness^3–5,76^. Finally, uncontrolled confounding factors—for example, changes in medication therapy or coexisting headaches, which were not systematically assessed—could have influenced the outcomes.

## Conclusion

The findings of this study underscore the important role of psychological factors in migraine-related disability. As postulated by the FAM, pain catastrophizing was identified as a relevant factor in cognitive-affective pain processing, and reductions in pain catastrophizing were associated with decreases in disability. Given its high prevalence in migraine, pain catastrophizing represents a potential therapeutic target that can be addressed through CBT-based interventions.

## Appendix

See the Table 8.

**Table 8:** Dropout: comparison of demographic and clinical baseline characteristics between completers and dropouts.

Characteristic	Completed, *n* = 55	Dropout, *n* = 73	*p*
Demographic characteristics			
Age, years, *M (SD) [range]*	36.1 (11.4) [18–61]	3 6.1 (12.1) [19–64]	0.853
Sex, *n (%)*			
Female	48 (87.3)	62 (84.9)	0.706
Male	7 (12.7)	11 (15.1)	
Migraine-related characteristics			
Mean monthly migraine days in the past 3 months, monthly, *M (IQR)*	8.2 (5.0–9.0)	7.4 (5.0–10)	0.725
Number of migraine days in the past 4 weeks, *M (IQR)*	8.0 (5.0–9.5)	7.6 (4.0–9.0)	0.753
Number of medication days in the past 4 weeks, *M (IQR)*	5.7 (4.0–6.8)	6.5 (4.0–9.0)	0.448
Mean pain intensity, *M (SD)*	5.5 (1.8)	5.7 (1.4)	0.732
Maximum pain intensity, *M (SD)*	7.5 (1.7)	7.3 (1.5)	0.414
Clinical characteristics			
HIT-6, *M (SD)*	66.4 (4.9)	65.4 (4.9)	0.368
PCS, *M (SD)*	25.6 (10.9)	26.4 (11.9)	0.620
PCS-3, *M (SD)*	9.4 (2.5)	9.1 (2.7)	0.652
FAMI, *M (SD)*	95.9 (22.9)	95.9 (19.3)	0.853
HMSE-G-SF, *M (SD)*	25.1 (7.6)	25.7 (6.9)	0.579
DASS depression, *M (SD)*	7.5 (5.5)	7.2 (5.4)	0.768
DASS anxiety, *M (SD)*	4.1 (3.7)	4.5 (4.4)	0.973
DASS stress, *M (SD)*	8.5 (4.1)	9.1 (5.0)	0.703

HIT-6, Headache impact test; DASS, depression anxiety and stress scales; PCS, pain catastrophizing scale; FAMI, fear of attacks in migraine inventory; HMSE-G-SF, headache management self-efficacy questionnaire (German short form); SD, standard deviation; IQR, interquartile range.

## Data Availability

The data of this study will be made available to other researchers upon reasonable request.
